# Energy-Efficient Fuzzy-Logic-Based Clustering Technique for Hierarchical Routing Protocols in Wireless Sensor Networks

**DOI:** 10.3390/s19030561

**Published:** 2019-01-29

**Authors:** Abdulmughni Hamzah, Mohammad Shurman, Omar Al-Jarrah, Eyad Taqieddin

**Affiliations:** 1 Department of Computer Engineering, Jordan University of Science and Technology, P.O. Box 3030, Irbid 22110, Jordan; ayhamzah128@cit.just.edu.jo (A.H.); aljarrah@just.edu.jo (O.A.-J.); 2 Department of Network Engineering and Security, Jordan University of Science and Technology, P.O. Box 3030, Irbid 22110, Jordan; alshurman@just.edu.jo

**Keywords:** WSN, fuzzy interface system, energy, efficiency, lifetime, clustering

## Abstract

In wireless sensor networks, the energy source is limited to the capacity of the sensor node’s battery. Clustering in WSN can help with reducing energy consumption because transmission energy is related to the distance between sender and receiver. In this paper, we propose a fuzzy logic model for cluster head election. The proposed model uses five descriptors to determine the opportunity for each node to become a CH. These descriptors are: residual energy, location suitability, density, compacting, and distance from the base station. We use this fuzzy logic model in proposing the Fuzzy Logic-based Energy-Efficient Clustering for WSN based on minimum separation Distance enforcement between CHs (FL-EEC/D). Furthermore, we adopt the Gini index to measure the clustering algorithms’ energy efficiency in terms of their ability to balance the distribution of energy through WSN sensor nodes. We compare the proposed technique FL-EEC/D with a fuzzy logic-based CH election approach, a *k*-means based clustering technique, and LEACH. Simulation results show enhancements in energy efficiency in terms of network lifetime and energy consumption balancing between sensor nodes for different network sizes and topologies. Results show an average improvement in terms of first node dead and half nodes dead.

## 1. Introduction

Wireless Sensor Networks (WSN) are applied in many fields such as in health-care, environmental sensing, and industrial monitoring [1,2,3,4]. A WSN is comprised of two sides: a Base Station (BS) and a number of distributed sensor nodes that interact with the environment by sensing some physical parameters. These sensors are required to perform sensing, initial data processing, and communication. The BS is tasked with receiving, processing, and providing data to the end user for decision making [5,6,7,8]. Nodes in WSN rely on their on-board, limited, non-rechargeable, and non-changeable batteries. Additionally, sensor nodes are limited in storage, memory, and CPU processing capabilities [6,7,8,9,10,11].

Because the sensor nodes and BS use wireless radio signals to exchange packets, energy-efficient routing protocols play a vital role in energy consumption and network lifetime [6,9,10,12,13]. Direct transmission to the BS consumes more energy than sending the same data over the same distance in multiple stages of shorter distances. Accordingly, clustering has received attention by researchers. Each node communicates directly with a Cluster Head (CH). In turn, the CH aggregates, compresses, and transmits the data to the BS or a neighbor CH [6,7,12,14,15,16].

Clustering algorithms can be classified into centralized or distributed approaches. In centralized approaches, the clustering algorithm utilizes the global knowledge of the network, while in the distributed approaches, local information is used to generate clusters. The Low Energy Adaptive Clustering Hierarchy (LEACH) protocol [12] is the first technique based on a probabilistic approach in CH election. Each node in the network generates a random number, and if this random number is less than a threshold value, the node assigns itself as the cluster head and broadcasts an advertisement message to all other nodes. Each of the receiving nodes (non-CHs) determines the CH to associate with based on the signal strength of the advertisement message.

Developing an energy-efficient communication protocol is a critical goal in WSN. Several energy-efficient routing protocols were proposed to address this issue. They all adopt the idea of clustering or chaining sensor nodes so the transmission to the BS occurs in multi-hops.

Clustering allows multi-hop transmission, data aggregation, data compression, and redundant data elimination. The benefits from clustering depend on the perfection of the clustering algorithm and the fitness of the exploited parameters. Unlike distributed clustering algorithms, which are performed by individual sensor nodes using their local information, the centralized clustering algorithms performed by the BS allow optimal clustering solutions, because the overall view of the WSN is available.

Many factors affect the clustering algorithms in WSN, for example the remaining energy in the sensor nodes and the distances from their BS. However, if the problem is carefully analyzed, other factors can be considered. Obtaining an optimal clustering solution requires scaling each parameter by a weight corresponding to its influence on the dissipated energy and network lifetime. Therefore, if the clustering algorithm exploits more energy-affecting factors, the clustering will be more efficient. The Fuzzy Inference System (FIS) is an efficient modeling tool to combine parameters for better parameter integration results.

We introduce a fuzzy-based centralized clustering technique for energy-efficient routing protocols in WSN. The proposed clustering technique uses fuzzy logic to elect CHs and enforces a separation distance between them for even CH distribution through the covered area. Separation distance is calculated adaptively based on the number of remaining live nodes, the dimensions of the area covered by these nodes, and the percentage of the desired CHs.

The proposed fuzzy model uses five parameters to prioritize opportunities of sensor nodes’ CH election. These factors are: the remaining energy of the sensor node, distance to the BS, density of surrounding sensor nodes (which are probable cluster members for the current sensor node if elected as a CH), compacting of surrounding sensor nodes, and finally, the location suitability calculated via the average of the local consumed energy for the surrounding nodes. As a secondary contribution to this research, we suggest adopting the Gini index [17] for energy balance evaluation among nodes in the WSN clustering algorithm.

The rest of this paper is organized as follows: In Section 2, we review the basics of WSNs and fuzzy logic. Section 3 provides the methodology road-map for the proposed approach. Section 4 presents the performance evaluation, which begins with the simulation settings, followed by the performance metrics, and ends with the results and discussion. Finally, Section 5 draws the conclusions of the entire study and discusses further potentials of follow-up research.

## 2. Literature Review

Hierarchical Routing Protocols (HRPs) for WSN were introduced in the literature for energy-efficient routing protocols. HRPs in WSN show higher energy and bandwidth efficiency over conventional routing protocols. Unlike flat routing protocols, where sensors transmit their data to the BS directly, HRPs allow sensors to transmit data via mediators. HRPs are either cluster based or chain based. In cluster-based HRPs, sensors are organized into clusters, and transmissions go through cluster heads, while in chain-based HRPs, sensors are organized as chains through which the transmissions pass [14].

LEACH [12] is the pioneering and the most referenced HRP; it achieves a tremendous performance regarding WSN useful lifetime and energy consumption balancing [18]. LEACH is a distributed cluster-based HRP that utilizes randomized rotation of CHs based on a probabilistic threshold to distribute the energy load evenly among the sensors in the network. LEACH-C (LEACH-Centralized) [19] is one of the most popular versions of LEACH; it is a cluster-based centralized approach in which the CH election and distribution over the WSN are controlled by the BS using simulated annealing. LEACH-C utilize nodes’ residual energies and positions from the BS [20]. LEACH-C has better performance over LEACH in useful network lifetime and energy dissipation [19,20].

The authors of TEEN [21] classify sensor networks as proactive or reactive networks based on their functional mode. In reactive mode, nodes respond immediately to the changes of relevant parameters of interest, while sensor nodes in proactive mode respond to the changes of relevant parameters of interest periodically. TEEN is an energy-efficient routing protocol for reactive WSN; it reduces unnecessary or redundant transmissions. TEEN outperforms existing conventional WSN protocols in energy efficiency.

Manjeshwar et al. [22] introduced APTEENas an extension to TEEN for both transmitting periodic data and reacting to time-critical situations [23]. APTEEN allows three types of queries: historical, on-time, and persistent, which are used in hybrid networks. Moreover, it introduces QoS requirements for the on-time queries by minimum delay using the TDMA schedule with a special time slot assignment [23].

HEED (Hybrid Energy-Efficient Distributed clustering) [6] periodically performs clustering of WSN and CH selection for each cluster based on nodes’ residual energy as a primary parameter and the proximity of a given node to its neighbors as a secondary parameter. HEED achieves uniform CH distribution across the network, increases network scalability and lifetime, and balances load on sensor nodes. Simulation results prove the effectiveness of HEED in prolonging network lifetime and supporting scalable data aggregation.

PEGASIS (Power-Efficient Gathering in Sensor Information Systems) [7] is a chain-based HRP, where each node communicates only with a close neighbor to reduce energy spent per round by transmitting to the BS in rounds. PEGASIS outperforms LEACH by 100–300% when 1%, 20%, 50%, and 100% of nodes die for different network sizes and topologies [7]. Cheng et al. [24] propose a way to confine the election range of CHs in LEACH by exploiting residual energy, the relative density of surrounding nodes, and centroid distance. Thereby, longer sensor nodes’ lifetime is achieved according to the authors.

A novel approach of adopting the *k*-means clustering algorithm in WSN routing protocols has been proposed in [25]. It achieves higher network lifetime over the existing LEACH and HEED, as their simulation results demonstrated. In [26], the authors proposed the Load-balancing Cluster-based Protocol (LCP) to increase network lifetime by the selection of the highest residual energy node in each round to be CH in each cluster. A modification to LEACH considering residual energy and node location to distribute CHs evenly was proposed in [27]. As the simulation results demonstrated, LEACH was improved by 40% in survival time. In [28], Min and Chun proposed a cluster-based HRP of two stages. In the first stage, the election of CHs is performed based on nodes’ residual energy and their relative position to the base satiation. In the second stage, the CHs forward the collected data to the BS indirectly via multi-hop forwarding, which prolongs network lifetime and saves energy, as proven by their simulation results.

Li et al. proposed a centralized WSN clustering approach based on Discrete Particle Swarm Optimization (DPSO) used in the context of the traveling sales-man problem [29]. In their approach, the BS collects status information about sensor nodes, then runs their own modified version of the DPSO algorithm to find the optimal topology for the WSN. This approach achieved 15% enhancement over LEACH in terms of WSN lifetime and showed higher ability to balance energy consumption among WSN nodes. In [30], the authors proposed a clustering technique for WSN based on applying a non-deterministic approach and adopting intra-phase and inter-phase clustering. In each phase of the non-deterministic approach, the optimal participation of clusters’ members in the process of selecting the corresponding CHs is enforced. This technique achieved enhancement of 42% over the standard LEACH in terms of WSN lifetime. In [31], a comparison study of genetic algorithm, differential evolution, and particle swarm optimization for efficient and fast WSN clustering was presented. In addition, the authors compared these techniques in terms of achieved fitness value. Based on their results, these techniques had significant improvements over LEACH in terms of network life-cycle and energy saving. A centralized clustering approach for HRP in WSN based on residual energy for sensor nodes, remaining energy variance, and coverage density was proposed in [32]. The proposed approach outperformed the existing protocols in terms of network lifetime.

Singh et al. [33] applied a prediction-based data reduction scheme for energy-efficient routing protocol design, where WSN is divided initially into grids, then CH election takes place. The authors in [10,11,15,16,34], Refs. [35,36,37,38,39,40,41,42,43,44,45,46,47] proposed fuzzy logic approaches for CH election in cluster-based HRPs. Each of these fuzzy logic-based approaches uses the partial combination of the parameters, residual energy, BS proximity, local distance, concentration, centrality, etc., to select CHs, but none of them uses an effective combination. Fuzzy logic-based clustering approaches proposed in the literature vary among centralized, distributed, and hybrid. However, most of them are centralized because fuzzy logic-based CH election requires high CPU cycles and high memory capacities. Furthermore, fuzzy logic-based clustering algorithms require global knowledge about sensors’ attributes, which would be costly in terms of energy and bandwidth if exchanged via the sensors themselves. Therefore, for fuzzy logic-based clustering in WSN, the centralized approaches are preferred [10,11].

Another way to design routing protocols for ad hoc networks and/or WSN is the cross-layer approach. Some examples of cross-layer protocols appeared in [48,49,50]. It has been observed that many of the cross-layer approaches rely on fuzzy logic. The authors in [51] proposed a fuzzy logic-based cross-layer routing algorithm for mobile ad hoc networks, while the research in [49] proposed a fuzzy logic-based cross-layer routing algorithm for WSNs.

The different classes of WSN routing protocols for single-layer and cross-layer are summarized in Table 1.

## 3. The Proposed Fuzzy Model and WSN Clustering Technique

In this section, we present the proposed fuzzy model used for CH election and a clustering technique based on the proposed fuzzy model to accomplish optimal clustering in WSN.

Different factors influence CH election in WSN. Therefore, they must be combined appropriately for the best decisions. FIS is an efficient mechanism for such a purpose. It allows combining all input parameters in such a way that reflects their effectiveness in CH election.

To achieve maximum benefits from fuzzy logic for CH election, it is necessary to explore the factors that have an impact on CH election, use effective means to measure each of these factors, and build an efficient fuzzy model characterized by the effective combination of fuzzy rules and the appropriate design for the fuzzy sets.

**Figure 1:**
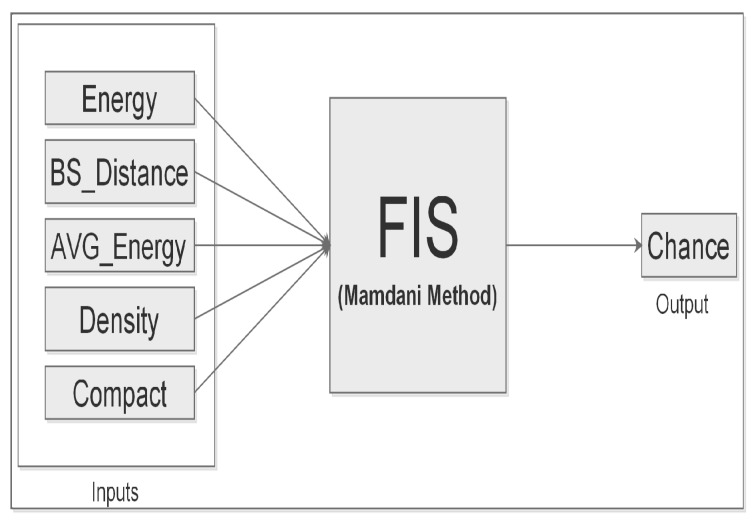
Fuzzy system control model.

Accordingly, the proposed FIS model scheme in Figure 1 is built to meet the above-mentioned requirements in order to achieve an efficient CH election in WSN.

### 3.1. Linguistic Variables’ Measurement and Normalization

The lifetime of the WSN is considerably influenced by the technique used for CH election, which in turn is influenced by many factors. These factors are expressed in the context of fuzzy logic as linguistic variables. Five linguistic variables are involved in the proposed fuzzy controller. They influence the network lifetime directly or indirectly by one of three aspects: energy consumed by CHs, total energy consumed by non-CH nodes (local consumed energy), or the distribution of energy consumption loads through sensor nodes.

The Min-Max normalization technique [53,54,55] (depicted in Equation (1)) is adopted for relative scaling of linguistic variables’ values. The values are calculated relative to a universe of discourse of zero and 100 according to their positions between minimum and maximum values. Finally, the sensor node values of a given variable are scattered along the universe of discourse according to their relative positions. Thus, the values of a given variable are normalized because they are required to assign the sensor node of maximum value with the highest priority to become a CH. The rest of the sensor nodes are prioritized according to their relative occurrences between the maximum and minimum variable values.

(1) $$Normalized\;\left(var\right)=\frac{Value(var)-Min(var)}{Max(var)-Min(var)}$$

where Value(var) is the given value of the given variable for the given current node and Min(var) and Max(var) are the minimum and maximum values of the given variable among all sensor nodes, respectively.

Note that in the calculation of any linguistic variable for a particular sensor node, any surrounding sensor nodes closer to a pre-selected CH must be excluded. This is because they will not become members of this node’s cluster in case it was elected.

**Figure 2:**
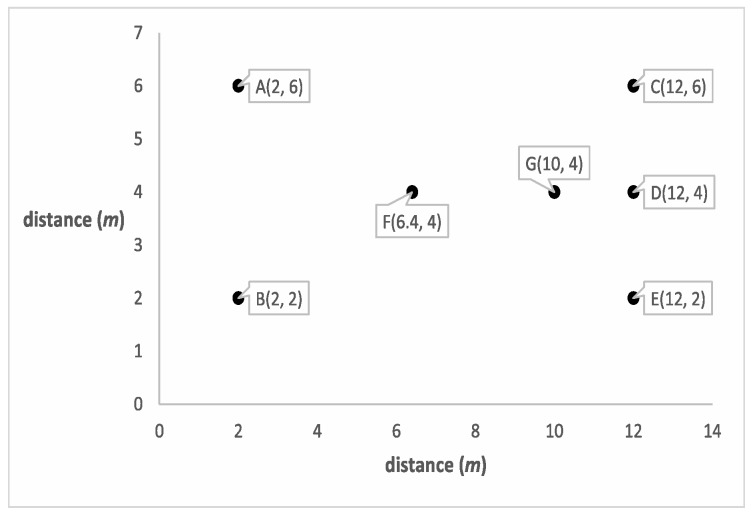
Simple WSN scenario for location suitability computation.

**Figure 3:**
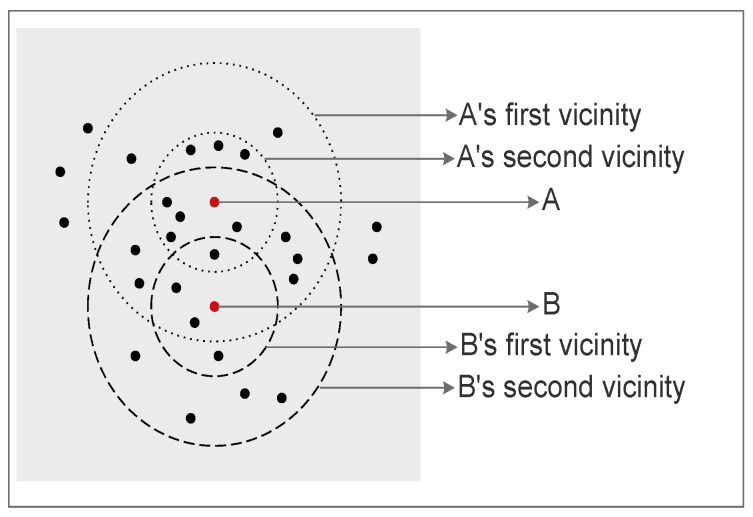
First and second vicinities of nodes A and B.

The following are the linguistic variables used in our proposed system:

1. **Remaining Energy:** Selecting sensor nodes with higher energy as CHs improves network lifetime by balancing energy consumption through the WSN’s nodes.
  Energy is normalized by Equation (1), where Value(var) is Energy of the current node and Max(var) and Min(var) are the maximum and minimum values among all candidate nodes, respectively. Hence, the normalized Energy will be zero for the node with the lowest remaining energy and 100 for the node with the highest remaining energy. The normalized Energy values for the rest of nodes are between zero and 100 according to their relative position between the highest and the lowest Energy values.
2. **Distance from the BS:** The lower the distance between CHs and the BS, the lower the consumed energy. Sensor nodes closer to the BS have to be given higher opportunities to be CHs over farther ones.
  *BS_Distance* is normalized as a percentage value to the distance between the furthest candidate node and the BS using Equation (1), where Max is the distance from the BS to furthest candidate node, Min is the distance of the nearest node to the BS, and Valueis the distance of current node from the BS.
  For example, if the BS_Distance of the farthest node from the BS is 80 m, the BS_Distance of the closest node to the BS is 30 m, and the BS_Distance of current node is 50 m, then the normalized BS_Distance of the current node is:
  $$Normalized\left(BS\_Distance\right)=\frac{50-30}{80-30}\times 100\%=40\%$$
3. **Location suitability:** This criterion measures how suitable a node location is as a CH with respect to surrounding nodes within a predefined range. A more suitable location for a CH node is a location with lower total communication energy.
  Location suitability for any node is measured by averaging the energy consumed locally by the sensor nodes located around the current node within a pre-defined range. We are interested in the nodes located within a predefined range as long as they are not closer in distance to any other pre-selected CH because they will be probably members of the current node if this current node wins CH election.
  Since we use the average of the consumed energies for the current node, we use the term *average of local consumed energy* and shorten it as AVG_Energy to point out the *location suitability*. Note that we measure the average of the consumed energy rather than the total consumed energy to guarantee that the size of the group does not have an effect on the normalized value.
  Some consider, mistakenly, that if a node has a lower sum of distances to its future probable members, then is situated at a more suitable location to become CH. This stems from the assumption that the nodes closer to the centroid of the group will consume less total energy. However, since the energy is exponentially proportional to the distance, it becomes necessary to consider members with extreme distances. This means that the location with the minimum average distances to other surrounding nodes in a predefined range is not always the most suitable location for a node to become a CH since it may not always result in minimum total consumed energy for its surrounding group of nodes.
  To illustrate this idea, consider the scenario depicted in Figure 2. Here, node G has the minimum average distance of 4.62488 m from its surrounding nodes, and is the closest to the centroid. If selected as a CH, it will result in an average consumed energy of $2.535168\times 10^{-7}$ J, for one byte of data from each neighboring node. On the other hand, node *F* has an average distance of 5.12655 m, but results in an average consumed energy of $2.529408\times 10^{-7}$ J. For that reason, we base the computation of location suitability for a node to become a CH on the consumed energy rather than the total distances.
4. **Density of surrounding nodes:** Selecting CHs surrounded by dense nodes over CHs surrounded by sparse nodes improves the energy consumption by increasing the opportunity for nodes with more neighbors in their vicinity to become CHs. Thereby, the local consumed energy for the group members is decreased.
  *Density* is calculated by the number of surrounding nodes within a predefined range normalized using Equation (1), where Value(var) is the density of the current node, Max(var) is the maximum density value through all candidate nodes, and Min(var) is the minimum density value through all candidate nodes.
5. **Compaction of surrounding nodes:** Group compaction is a measure of how the surrounding nodes are distributed around the current node. A node surrounded by more neighbors in a closer vicinity is considered of higher *compaction*degree. Selecting a node with a higher compactiondegree minimizes the total energy consumption. This criterion is important to assign different priorities for CH candidates with the same number of surrounding nodes within a predefined range. In other words, it is beneficial for distinguishing candidates surrounded by the same density of sensor nodes.
  *Compaction* is calculated as the ratio of the number of nodes located within the first vicinity region to those located within the second vicinity region. The first vicinity region is that region that is within half of a predefined radius distance, whereas the second vicinity region is the one that is within the nodes’ radius.
  As shown in Figure 3, the small circles surrounding A and B are considered as the first vicinities, and the larger ones are considered as the second vicinities for nodes A and B. The distance to which to extend the first vicinity is a design parameter and may be any fraction of the radius.
  To normalize the *Compaction*, we use Equation (1), where Value(var) is the *Compaction* value of current node, Max(var) is the maximum *Compaction* value through all candidate nodes, and Min(var) is the minimum *compaction* value through all candidate nodes.

### 3.2. Fuzzy Sets

Each of the linguistic variables is divided into overlapping fuzzy sets, called membership functions. The crisp value of the linguistic variable belongs to each of the linguistic variable fuzzy sets with a different degree of membership. The number of membership functions and their overlaps for each of the inputs/output linguistic variables are taken based on the trial-and-error method over tens of experiments.

**Figure 4:**
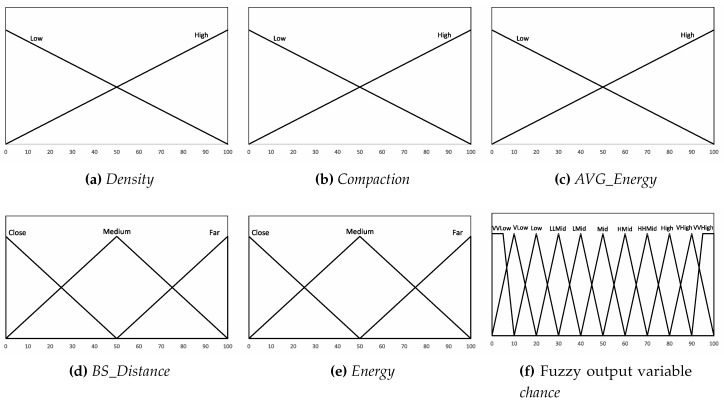
Membership Functions of the fuzzy sets.

In this section, we present membership functions for each of the five input linguistic variables used and the output linguistic variable *chance* in Figure 4. Since each of the input parameters affects the consumed energy and the WSN lifetime to a different extent, the Mamdani inference model rules are formulated to reflect this nature of relationship. We ran tens of experiments to explore a better construction of rules using the trial-and-error method. The rules’ combinations are shown in Table 2.

### 3.3. The Proposed Clustering Technique FL-EEC/D

The *FL-EEC/D* technique uses the aforementioned fuzzy model for CH election. It controls the distribution of CHs based on determining and enforcing a specific minimum separation distance between CHs to guarantee their fair distribution. Each CH must be far from the closest CH by the distance *d*, as a minimum. The distance *d* is adaptive depending on the dimensions of the WSN, the number of nodes, and the desired CHs percentage. It is computed using Algorithm 1.

**Algorithm 1** Calculation of the minimum forced distance between CHs.**function**Calc_Min_Dist(N,p,dimX,dimY)*N*: List of live nodes*p*: percentage of CHs in WSNdimX: length of X-dimension, dimY: Length of Y-dimension    cc ← ceiling(p×count(N))cc: desired # of clusters    *c* ← 1*c*: # of of regional columns, initially set to 1    *r* ← 1*r*: # of regional rows, initially set to 1    df ← cc−1df: difference between # of created rectangles and the desired # of clusters, initially set to cc−1    *I* ← 1    **while**
$I\leq ceiling(\sqrt{cc})$
**do**        *J* ← *I*        **while**
J≤I+1
**do**            **if**
df≥|cc−(I×J)|
**then**                cc ← |cc−(I×J)|                *c* ← *I*                *r* ← *J*            **end if**            *J* ← J+1        **end while**        *I* ← I+1    **end while**    rX ← dimX    rY ← dimY    *d* ← $\frac{\sqrt{rX^{2}+rY^{2}}}{2}$    **return**
*d**d*: minimum distance between CHs
**end function**


Firstly, Algorithm 1 virtually divides the WSN into identical regions that are aligned into rows and columns resembling the shapes of rectangles. For the purpose of keeping the shapes of these rectangles compacted, the dimensions of the rectangles are set to be as equal as possible. Therefore, the difference between the number of rows and columns is not allowed to be more than one. The domain for the allowed number of rectangles is the set of square numbers and the numbers that are products of two successive numbers. This set is defined as:

$$K=\{\left(p_{i}\times p_{i}\right),\left(p_{i}\times p_{i+1}\right);i\geq 1,p\geq 1\}$$

In case the desired number of CHs does not belong to the set *K*, then it will be changed to the nearest number within the set *K*. For example, if the required number of CHs is three, then the resulting number of rectangles is either two or four. The number three does not belong to the set *K* because it is neither a square number, nor a number that can be factored into two successive numbers. However, the number two can be factored into the successive numbers two and one, and the number four is a square number.

**Figure 5:**
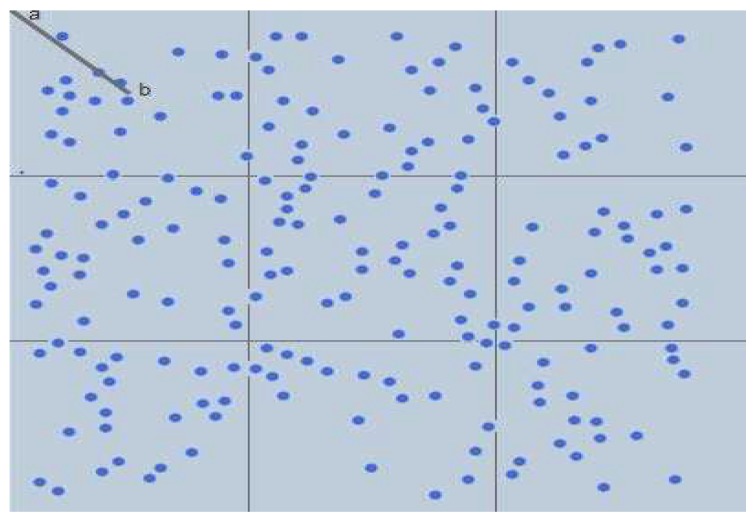
WSN area divided into virtual identical rectangles.

Since the rectangles are aligned in rows and columns, their total number does not always match exactly the number of the desired clusters. The algorithm continues to add new columns in each time until it creates a number of rectangles equal or as close as possible to the desired number of clusters. The algorithm also adds a new row in each iteration before adding a new column to keep the number of rows as equal as possible to the number of columns. After that, the algorithm takes half of the diagonal of any rectangle as the forced minimum separation distance between CHs, as shown in the first rectangle by line |ab| in Figure 5.

The algorithm initially considers the whole WSN as a single rectangle, initializing the number of column and rows to one. In each round of the outer loop of the algorithm, a new column of rectangles is added. Furthermore, in each round of the inner loop, a new row of rectangles is added, where the resulting number of rectangles is closer to the desired number of clusters. The outer loop continues to loop as long as the number of columns is less than or equal to the square root of the desired number of clusters, while the inner loop is responsible for adding rows loops twice for each outer loop round.

**Figure 6:**
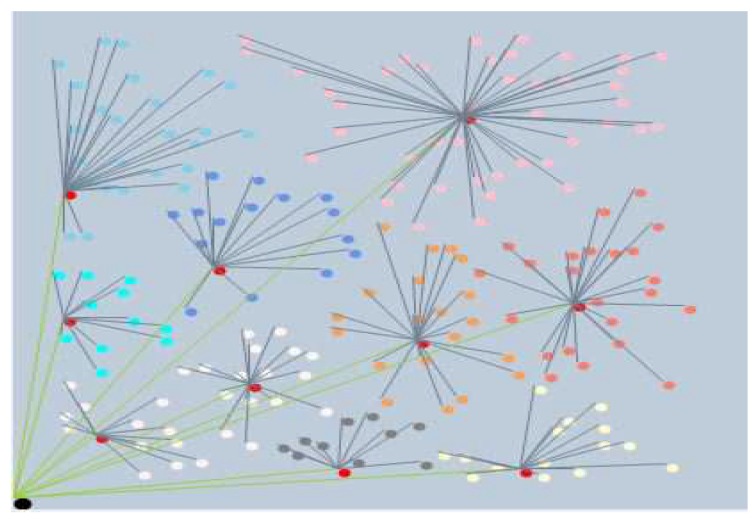
A snapshot of clustering output in *FL-EEC/D*.

The clustering scheme is based on forcing a minimum separation distance between CHs. It begins by selecting CHs using the proposed fuzzy inference model explained previously on the basis of separating CHs by distance *d* and ends by forming clusters by mapping each node $n_{i}$ to the closest $CH_{r}$. Algorithm 2 provides the detailed steps. Figure 6 shows a snapshot of clustering output in *FL-EEC/D*.

**Algorithm 2** Clustering based on minimum separation distance enforcement between CHs.
**procedure**
WSN clustering
    *inputs*:    $N=\{n_{1},n_{2},\ldots ,n_{k}\}$
% N: set of live nodes %    range ← *d*% *d*: minimum forced distance %    *p*% *p*: Percentage of live nodes to become CHs %    dc ← Ceiling(p×count(N))% dc: number of the desired CHs %    *outputs*:    $CH=\{CH_{1},CH_{2},\ldots ,CH_{dc}\}$% set of CHs %    $C=\{C_{1},C_{2},\ldots ,C_{dc}\}$% set of clusters %    *steps:*    1) For each node $n_{i}\in N$, if the distance from $n_{i}$ to the closest already selected CH is less than *d*, then calculate each of the fuzzy input variables: *Energy,* BS_Distance, Density, Compaction AVG_Energy.    2) Calculate the fuzzy output variable *chance* for each $n_{i}\in N$ based on the linguistic variables using the proposed fuzzy inference system    3) Select $n_{i}$ with the highest *chance* value among the nodes located away from any pre-selected CH by the distance *d*    4) Repeat Steps 1–4 until reaching dc    5) Form clusters, $C=\{C_{1},C_{2},\ldots ,C_{dc}\}$, by joining each $n_{i}\in N$ to the closest $CH_{r}\in CH$
**end procedure**


## 4. Performance Evaluation

We implemented the algorithms in *.NET* using the *FuzzyLite* library. It is used to simulate and evaluate comparatively the efficiency of the proposed approach in terms of lifetime, overall remaining energy, and energy balancing against the well-known clustering algorithm LEACH [12] and against approaches proposed in [34] and [25].

In [34], the authors proposed a fuzzy logic model for CH election in WSN; while the approach proposed in [25] is based on the well-known *K*-means clustering algorithm. In this work, we refer to them as *FL[1]/D* and reference the approach proposed in [34] as *K-means-LEACH*; respectively.

### 4.1. Simulation Parameters

We adopt the first order model [12] for the simulations. Here, the energy consumed for sending a *k*-bit message for a distance *d* is calculated as:

(2) $$E_{Tx}\left(K,d\right)=K\left(E_{elec}+\varepsilon_{amp}\times d^{2}\right)$$

where $E_{amp}$ is the energy consumed by the amplifier circuit to send one bit for a distance of one meter. $\varepsilon_{amp}$ can be expressed as $\varepsilon_{fs}$ or $\varepsilon_{mp}$ according to the distance between the source node and the destination node.

For the simulations, the nodes are randomly distributed over the area of the WSN. The sensors are connected to their BS via a single level of CHs. Each node sends five packets per round, with each packet containing 500 bytes.

Table 3 shows the common simulation parameters for the WSN through the simulation experiments [56].

### 4.2. Network Model

We make the following assumptions in evaluating the proposed approach.

1. All sensor nodes are randomly distributed over a two-dimensional area.
2. All sensor nodes are homogeneous in terms of processing and communication capabilities. Furthermore, they have the same battery, radio, sensing, and storage capabilities.
3. There are no recharging capabilities.
4. The BS is able to estimate the locations of the sensor nodes by using any localization technique. This may be based on utilizing GPS [57,58] or by adopting GPS-free localization such as weighted centroid localization [59,60,61], which is based on the received signal strength. Generally, a small error in localization will not be significant for the overall clustering result since the values are calculated as relative variances between the lowest and highest values. These relative variances are later fuzzified (expressed as linguistic variables). The percentage of cluster heads is supposed to be 5% of the total number of sensor nodes in the WSN.
5. Cluster heads are re-selected periodically.

### 4.3. Performance Metrics

We considered the metrics of lifetime and total consumed energy to evaluate the schemes and methods proposed by this research comparatively. However, using the total consumed energy per round to measure energy efficiency may not be an accurate measure for lifetime evaluation. This is because the total energy might be maintained by a small percentage of nodes, while all other nodes were depleted or the total maintained energy might be less, but is distributed over a larger percentage of nodes. In order to judge the energy efficiency of the WSN clustering technique accurately, we propose a way to measure how much the clustering technique can maintain of the remaining total energy distributed equally through the nodes. If it manages to keep the total remaining energy distributed more equally, then more nodes will be live for further rounds. To the best of our knowledge, this metric of equality for the distribution of remaining energy through the nodes is used here for the first time. Furthermore, we use the well-known Gini index as a means to measure this property.

**Figure 7:**
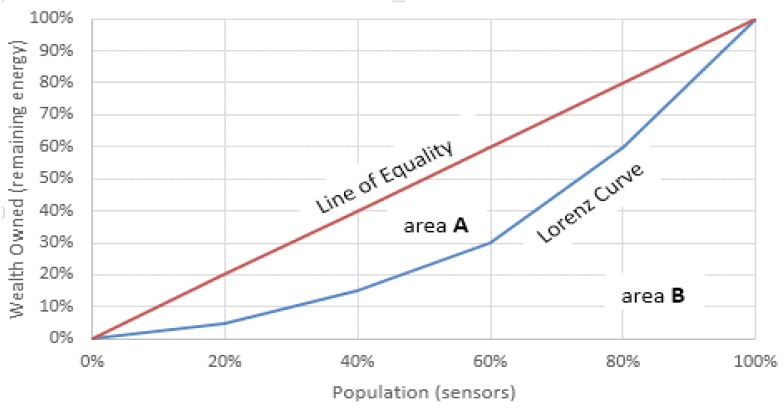
Lorenz curve and the line of equality for the distribution of wealth.

The Gini index is used in the context of measuring the extent of the inequality of income among the population or to measure how unequally the resources are distributed among population samples. The Gini index value is between zero and one; a value of zero means the inequality of the measured resource among all samples is zero, i.e., the resource is distributed equally among population samples, which happens when all samples have the same amount of resource; while a Gini index value of one means that the inequality of resources among population samples is at the maximum, when all of the resources are possessed by one sample only. In general, the larger the value of the Gini index, the more unequal the resources among the population samples, and vice versa. Mathematically, the Gini coefficient is defined as the ratio of area confined between the wealth distribution curve, Lorenz curve, and the line of equality, area A, to the area under the line of equality, area A+B, in Figure 7.

The Lorenz curve is the accumulative percentage of people against the accumulative share of wealth [62]. However, in the context of this research, the population is the sensor nodes, and the remaining energy is used in place of wealth. We adopt it to measure the extent to which the clustering algorithm keeps the remaining energy distributed equally among the nodes. Thus, the smaller the Gini index, the more equal the distribution of the remaining energy.

We also measure the network lifetime in terms of the First Node Dead (FND), 10% of Nodes Dead (10PND), Quarter of Nodes Dead (QND), Half of Nodes Dead (HND), and 75% of nodes dead.

### 4.4. Evaluation of FL-ECC/D

A comparative evaluation of FL-EEC/D is performed using many scenarios, to illustrate and validate its behavior under different densities, sparse, moderate, or dense, and through different positions of the BS. The comparison is based on the metric of energy balancing and network lifetime in terms of FND, 10PND, QND, and HND. All nodes in the scenarios are randomly distributed over an area of 200×200 meters.

**Figure 8:**
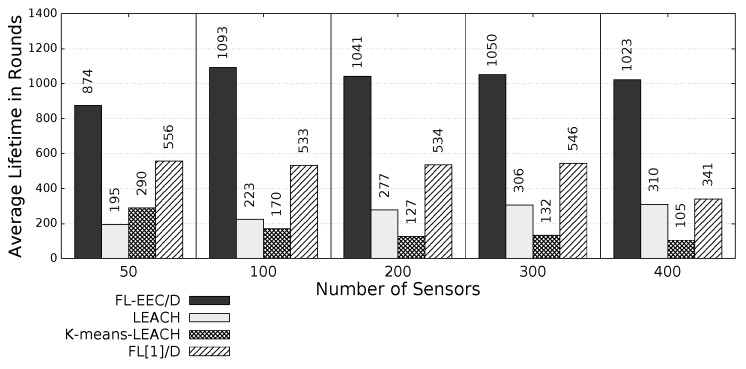
FND of FL-EEC/D against LEACH, *K*-means-LEACH, and FL[1]/D (BS at the center of WSN).

**Figure 9:**
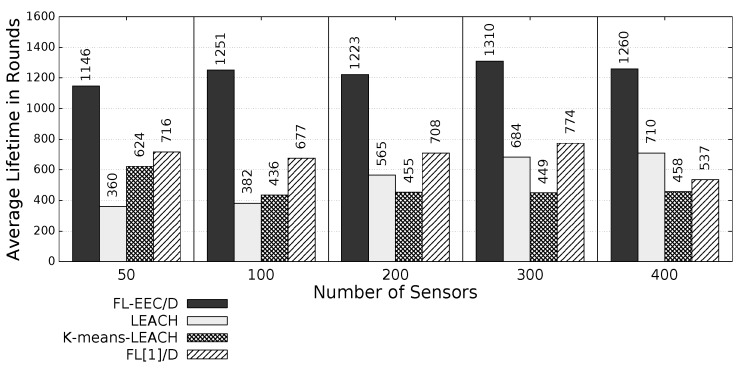
10PND of FL-EEC/D against LEACH, *K*-means-LEACH, and FL[1]/D (BS at the center of WSN).

**Figure 10:**
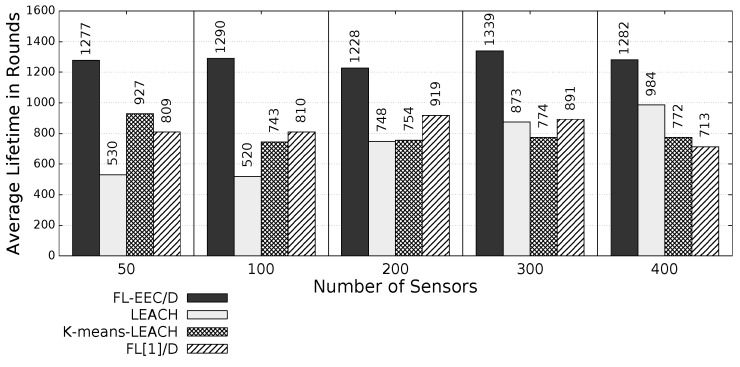
QND of FL-EEC/D against LEACH, *K*-means-LEACH, and FL[1]/D (BS at the center of WSN).

**Figure 11:**
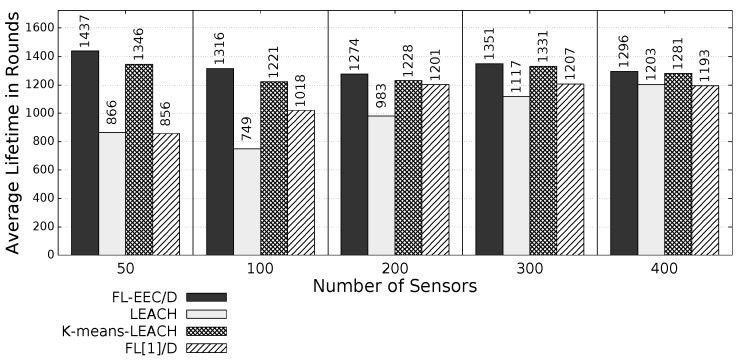
HND of FL-EEC/D against LEACH, *K*-means-LEACH, and FL[1]/D (BS at the center of WSN).

The proposed *FL-EEC/D* is comparatively evaluated for the case of positioning the BS at the center of WSN. Figure 8, Figure 9, Figure 10 and Figure 11 compare the achieved average lifetime of FL-EEC/D for FND, 10PND, QND, and HND, respectively, against the achieved average lifetime of LEACH, FL[1]/D, and *K*-means-LEACH. The network sizes are 50,100,200,300, and 400 nodes.

It is clear from these four figures that the proposed FL-EEC/D achieved a longer average of network lifetime for each of the terms FND, 10PND, QND, and HND, for all network sizes. For example, considering Figure 8, we see that the network lifetime achieved by FL-EEC/D in terms of FND for the network of 50 nodes was approximately 4.48-, 3-, and 1.6-times the average of that achieved by LEACH, *K*-means-LEACH, and FL[1]/D, respectively.

**Figure 12:**
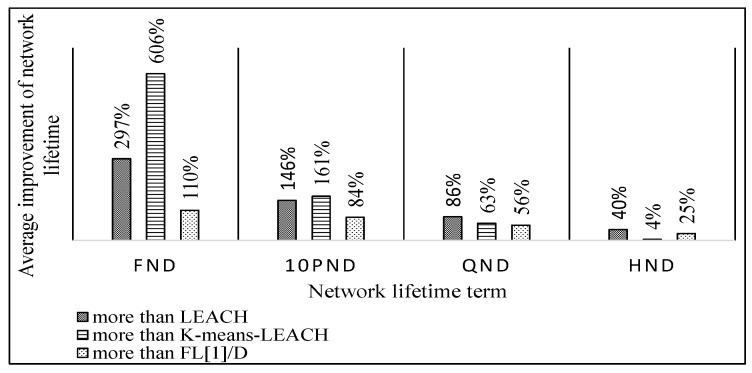
Average improvement percentage of FL-EEC/D over LEACH, *K*-means-LEACH, and FL[1]/D for network lifetime (BS at the center of WSN).

Figure 12 demonstrates the overall achieved enhancement of the proposed FL-EEC/D against LEACH, FL[1]/D, and *K*-means-LEACH for the network lifetime metric in terms FND, 10PND, QND, and HND, for the five WSN sizes. The average improvement diminishes as the rounds progress, and this indicates that we are achieving a good balance in the distribution of energy an extending the time before the events of FND, 10PND, and QND occur.

**Figure 13:**
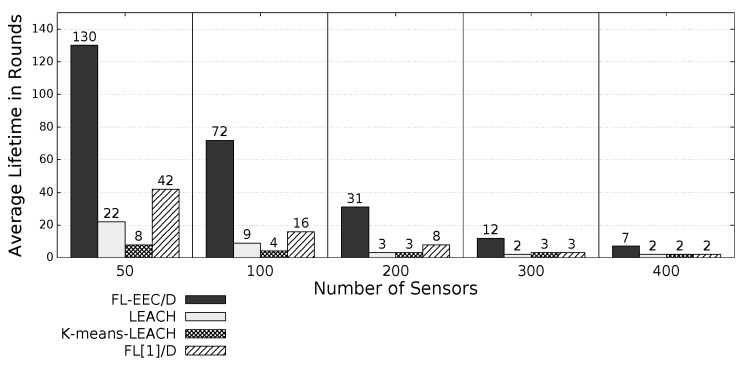
FND of FL-EEC/D against LEACH, *K*-means-LEACH, and FL[1]/D (BS at the corner of WSN).

**Figure 14:**
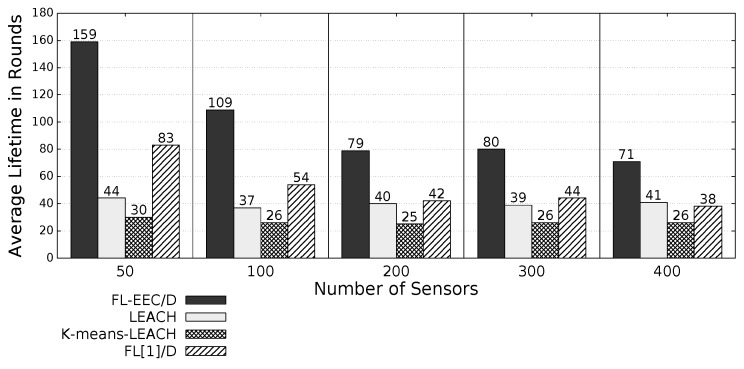
10PND of FL-EEC/D against LEACH, *K*-means-LEACH, and FL[1]/D (BS at corner of WSN).

**Figure 15:**
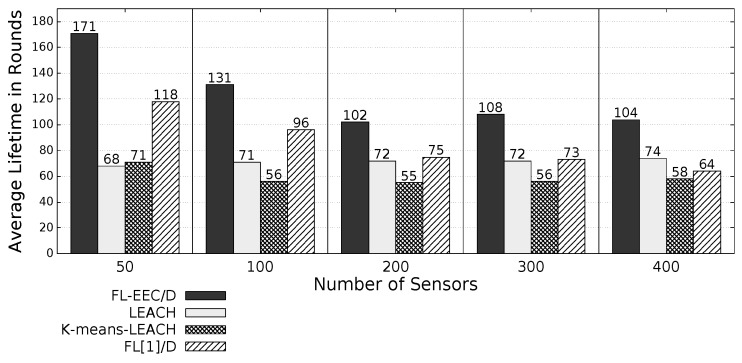
QND of FL-EEC/D against LEACH, *K*-means-LEACH, and FL[1]/D (BS at the corner of WSN).

**Figure 16:**
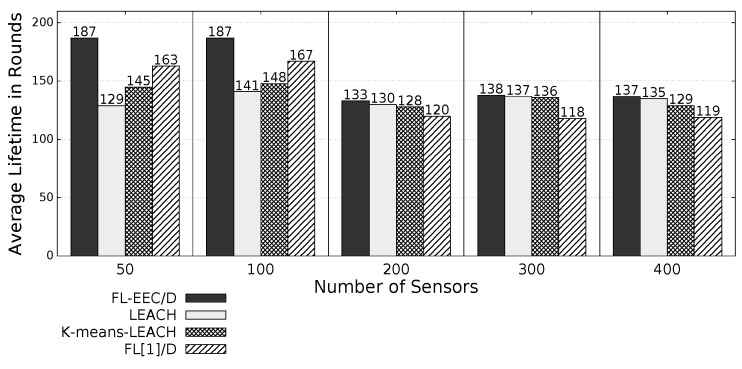
HND of FL-EEC/D against LEACH, *K*-means-LEACH, and FL[1]/D (BS at the corner of WSN).

We further evaluate the proposed *FL-EEC/D* for the same metrics, but change the position of the BS to the corner of the WSN. Figure 13, Figure 14, Figure 15 and Figure 16 present the achieved average network lifetime in terms of FND, 10PND, QND, and HND, respectively, for the different WSN sizes.

**Figure 17:**
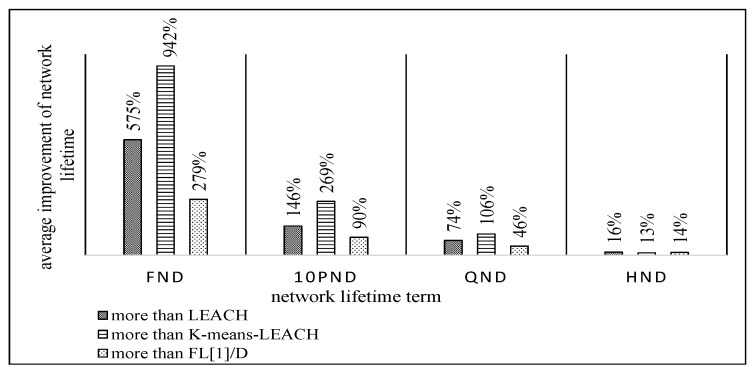
Average improvement percentage of FL-EEC/D over LEACH, *K*-means-LEACH, and FL[1]/D for network lifetime (BS at the corner of WSN).

It is obvious from these figures that FL-EEC/D highly outperformed its counterparts. However, placing the BS at a farther position resulted in a faster depletion of the nodes, for all schemes. Here, the advantage of FL-EEC/D is more prevalent, as its enhancement over the other schemes became higher, as demonstrated in Figure 17.

The improvements achieved by the FL-EEC/D scheme point to the ability of balancing the energy through the nodes. This is a result of better selection of the CHs and interchanging the load over the nodes in a more balanced approach.

As shown in Figure 12 and Figure 17, the improvement of network lifetime starts high for FND and then gradually decreases for 10PND, QND, and HND, respectively. This sequence is evidence of the strength of collaboration between different sensors to take loads of CH operations. Thus, most of the nodes operate together for the longest possible duration and then almost die together. In other words, they tend to die in groups rather than individually. This is contrary to the case of less balancing of remaining energy between different sensors.

**Figure 18:**
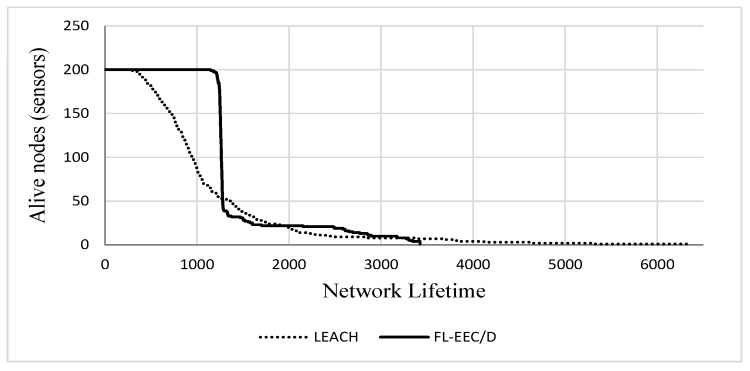
Live nodes vs. rounds for FL-EEC/D and LEACH.

Referring to Figure 18, we see that the line representing the live nodes of FL-EEC/D takes the form of a step function with a sudden drop, while the line representing the live nodes of LEACH tends to decrease gradually, and the drops are larger in the earlier period of network lifetime. Thus, most of the nodes in LEACH die through the earlier period of network lifetime. In contrast, the FL-EEC/D overcomes this shortcoming and always works to prolong sensors lifetime when it is worthy to keep them alive.

To analyze the energy consumption in FL-EEC/D versus the other techniques, we take a WSN consisting of 200 nodes distributed randomly over an area of 200×200 meters using the same simulation parameters listed in Table 3. The BS is located in the area at position (100,100). The efficiency of managing energy by minimizing total consumed energy and balancing it among the sensors is the idea behind prolonging the network lifetime. Moreover, as observed earlier, minimizing the total consumed energy without balancing the energy reserves of the nodes does not necessarily result in better network lifetime. It is more important to minimize the differences between remaining energies among the different nodes.

**Figure 19:**
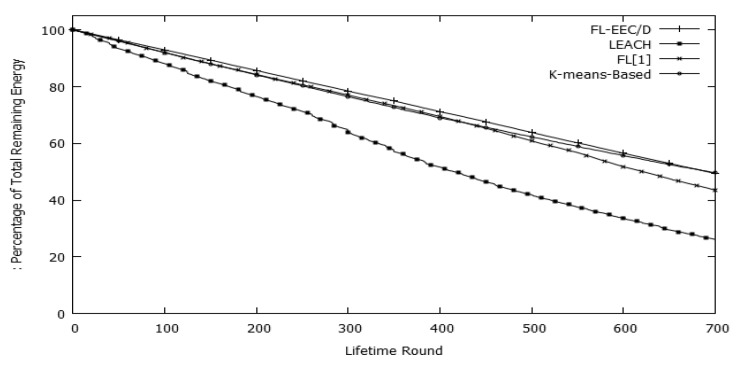
Percentage of total remaining energy per round for FL-EEC/D, LEACH, *K*-means-LEACH, and FL[1]/D.

Figure 19 depicts the percentage of total remaining energy for the rounds before HND. The figure shows that the FL-EEC/D conserves more total energy than the other three schemes. It also shows that the FL-EEC/D tends to consume energy gradually per round in equal amounts.

**Figure 20:**
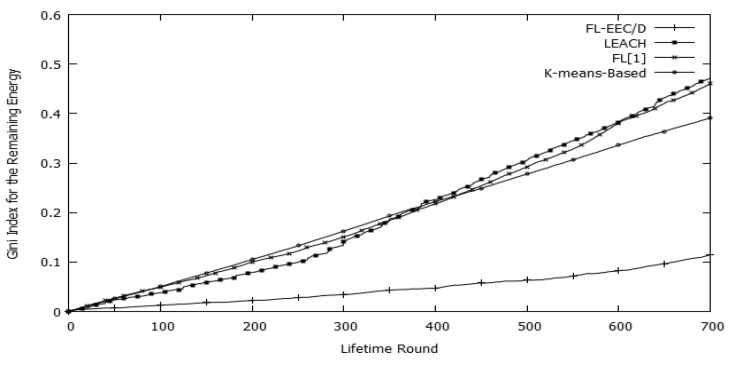
The Gini Index of the remaining energy per round for FL-EEC/D, LEACH, *K*-means-LEACH, and FL[1]/D.

Figure 20 shows that the proposed FL-EEC/D achieves significantly lesser Gini index values for the remaining energy via all the rounds before HND occurrence. This means that the FL-EEC/D keeps the remaining energy among the nodes more equally balanced compared to the three other protocols.

## 5. Conclusions and Future Work

FISs are the best choice for building effective clustering algorithms/techniques for energy-efficient routing protocols in WSN, due to its high ability of combining and effectively blending input parameters to produce proper decisions about CH selections.

To achieve the best possible results of energy-efficient routing protocols in WSN, it is recommended to utilize every parameter having an effect on the energy efficiency of the WSN routing protocol. Furthermore, it is recommended to integrate them in a way that reflects the extent to which each affects the energy efficiency of the WSN. In this work, we introduced the FL-EEC/D clustering technique for energy-efficient routing protocols. Furthermore, we proposed an efficient fuzzy logic used by this clustering technique to perform CH election. This fuzzy logic utilizes five parameters to determine the strength of each sensor’s chance to be a CH. These parameters are: remaining energy of the given sensor node, distance of sensor nodes from the BS, density of other surrounding sensor nodes around the candidate CH, compaction of nodes around the sensor node, and the average of the local consumed energy. We set a condition to control the distribution of CHs over the WSN area by forcing an adaptive minimum separation distance between CHs to guarantee their even distribution. FL-EEC/D was comparatively evaluated by simulating various WSN scenarios against LEACH [12], *K*-means-LEACH [25], and FL[1]/D [34] for the metrics of total energy consumption, energy balancing, and network lifetime in terms of FND, 10PND, QND, and HND. Simulation results show that FL-EEC/D significantly outperforms these approaches in the metrics of network lifetime and energy consumption efficiency in various simulated scenarios. Furthermore, we introduced the idea of adopting the Gini index measurement mean for measuring the extent to which the WSN clustering algorithm has the ability to balance energy consumption through all WSN sensor nodes. We used the Gini index as a fair measurement tool for evaluating the energy efficiency of routing protocols in WSN for the metric of balancing of energy distribution.

## Figures and Tables

**Table 1:** Classes of WSN routing protocols.

Class		Single Layer	Cross Layer
**Cluster Based**	**Centralized**	Sensors are organized into clusters, and transmissions go through CHs. In this approach, node locations are estimated by the BS. This benefits from the global knowledge of the network and achieves a more efficient clustering compared to the distributed approach. Examples appear in [19,25,26,29], and [32].	Information is collected and combined from different network layers to be utilized for CH election. The works in [49] and [51] follow this approach.
	**Distributed**	Sensors are organized into clusters in a distributed manner, and transmissions go through CHs. A drawback of this approach is that it is confined to local information, and this may lead to non-optimal WSN clustering [6,12,24,27,52].	
**Chain Based**	Proactive	Sensors are periodically organized into chains through which the transmissions pass. An example of this approach is found in [7].	
	Reactive	Sensors are organized as chains through which the transmissions pass. Routes change immediately in response to the changes of relevant parameters of interest [21].	

**Table 2:** Fuzzy rules.

SN.	Input Variables					Output Variable
	Energy	BS_Distance	AVG_Energy	Density	Compaction	Chance
1	Low	Close	Low	Low	Low	LMid
2	Low	Close	Low	Low	High	HMid
3	Low	Close	Low	High	Low	HMid
4	Low	Close	Low	High	High	High
5	Low	Close	High	Low	Low	Low
6	Low	Close	High	Low	High	LMid
7	Low	Close	High	High	Low	LMid
8	Low	Close	High	High	High	HMid
9	Low	Medium	Low	Low	Low	VVLow
10	Low	Medium	Low	Low	High	VLow
11	Low	Medium	Low	High	Low	VLow
12	Low	Medium	Low	High	High	LLMid
13	Low	Medium	High	Low	Low	VVLow
14	Low	Medium	High	Low	High	VVLow
15	Low	Medium	High	High	Low	VVLow
16	Low	Medium	High	High	High	VLow
17	Low	Far	Low	Low	Low	VVLow
18	Low	Far	Low	Low	High	VVLow
19	Low	Far	Low	High	Low	VVLow
20	Low	Far	Low	High	High	VVLow
21	Low	Far	High	Low	Low	VVLow
22	Low	Far	High	Low	High	VVLow
23	Low	Far	High	High	Low	VVLow
24	Low	Far	High	High	High	VVLow
25	Medium	Close	Low	Low	Low	High
26	Medium	Close	Low	Low	High	VVHigh
27	Medium	Close	Low	High	Low	VVHigh
28	Medium	Close	Low	High	High	VVHigh
29	Medium	Close	High	Low	Low	HMid
30	Medium	Close	High	Low	High	High
31	Medium	Close	High	High	Low	High
32	Medium	Close	High	High	High	VVHigh
33	Medium	Medium	Low	Low	Low	LLMid
34	Medium	Medium	Low	Low	High	Mid
35	Medium	Medium	Low	High	Low	Mid
36	Medium	Medium	Low	High	High	HHMid
37	Medium	Medium	High	Low	Low	VLow
38	Medium	Medium	High	Low	High	LLMid
39	Medium	Medium	High	High	Low	LLMid
40	Medium	Medium	High	High	High	Mid
41	Medium	Far	Low	Low	Low	VVLow
42	Medium	Far	Low	Low	High	VVLow
43	Medium	Far	Low	High	Low	VVLow
44	Medium	Far	Low	High	High	Low
45	Medium	Far	High	Low	Low	VVLow
46	Medium	Far	High	Low	High	VVLow
47	Medium	Far	High	High	Low	VVLow
48	Medium	Far	High	High	High	VVLow
49	High	Close	Low	Low	Low	VVHigh
50	High	Close	Low	Low	High	VVHigh
51	High	Close	Low	High	Low	VVHigh
52	High	Close	Low	High	High	VVHigh
53	High	Close	High	Low	Low	VVHigh
54	High	Close	High	Low	High	VVHigh
55	High	Close	High	High	Low	VVHigh
56	High	Close	High	High	High	VVHigh
57	High	Medium	Low	Low	Low	HHMid
58	High	Medium	Low	Low	High	VHigh
59	High	Medium	Low	High	Low	VHigh
60	High	Medium	Low	High	High	VVHigh
61	High	Medium	High	Low	Low	Mid
62	High	Medium	High	Low	High	HHMid
63	High	Medium	High	High	Low	HHMid
64	High	Medium	High	High	High	VHigh
65	High	Far	Low	Low	Low	Low
66	High	Far	Low	Low	High	LMid
67	High	Far	Low	High	Low	LMid
68	High	Far	Low	High	High	HMid
69	High	Far	High	Low	Low	VVLow
70	High	Far	High	Low	High	Low
71	High	Far	High	High	Low	Low
72	High	Far	High	High	High	LMid

**Table 3:** Simulation parameters.

Parameter	Value
Data Packet Size	500 bytes
Initial Energy	2 J
$E_{elec}$	50 nJ/bit
$\varepsilon_{fs}$	10 nJ/bit/m${}^{2}$ bytes
$E_{fus}$	5 nJ/bit/signal
$\varepsilon_{mp}$	0.0013 nJ/bit/m${}^{4}$
Threshold Distance $d_{0}$	87 m
Deployment Method	Random

## Abbreviations

The following abbreviations are used in this manuscript:

WSN	Wireless Sensor Network
CH	Cluster Head
BS	Base Station
FL-EEC/D	Fuzzy Logic-based Energy-Efficient Clustering based on minimum separation Distance enforcement between CHs
LEACH	Low Energy Adaptive Clustering Hierarchy
FIS	Fuzzy Inference System
HRP	Hierarchical Routing Protocols
PEGASIS	Power-Efficient Gathering in Sensor Information Systems
DPSO	Discrete Particle Swarm Optimization
FND	First Node Dead
10PND	10% of Nodes Dead
QND	Quarter of Nodes Dead
HND	Half of Nodes Dead
